# Pulmonary Tumor Embolism: A Rare Cause of Acute Pulmonary Hypertension

**DOI:** 10.7759/cureus.11877

**Published:** 2020-12-03

**Authors:** Timothy Chong, Joseph Park, Hafiz M Aslam, Shahryar Ansari, Sara L Wallach

**Affiliations:** 1 Internal Medicine, Drexel College of Medicine, Philadelphia, USA; 2 Hematology and Medical Oncology, East Carolina University, Greenville, USA; 3 Internal Medicine, St. Francis Medical Center, Seton Hall University, Trenton, USA; 4 Internal Medicine, Hackensack Meridian School of Medicine at Seton Hall University, Trenton, USA

**Keywords:** pulmonary tumor embolism

## Abstract

A rare cause of acute decompensated pulmonary hypertension is pulmonary tumor embolism (PTE), which is an uncommon complication of advanced lung malignancy. Patients diagnosed with PTE typically have a poor prognosis, and so patients with advanced lung tumors who present with signs of right heart failure and respiratory support should be evaluated for PTE. We present a case of a 54-year-old Hispanic female who initially presented with a one-month history of dysphagia, who was found to have acute pulmonary hypertension secondary to invasion of the pulmonary arteries by lung adenocarcinoma.

## Introduction

Acute decompensated pulmonary hypertension presents with signs of rapidly progressive right heart failure which can lead to multisystem organ failure. Some triggers that are thought to cause acute pulmonary hypertension include arrhythmias, infections, or thromboembolic events [1]. Thromboembolism causes mechanical obstruction of pulmonary vasculature, resulting in pulmonary hypertension. In rare instances, other pathologies such as malignancies can occlude the pulmonary arteries [2]. We present a case of massive pulmonary tumor embolism (PTE) causing acute pulmonary hypertension and cor pulmonale in a patient with recurrence of stage IV lung adenocarcinoma with metastases to the spine.

## Case presentation

A 54-year-old Hispanic female initially presented to the hospital with a one-month history of dysphagia. Her past medical history is significant for stage IV adenocarcinoma of the right lung with metastases to the L2 spine in remission status post radiotherapy and chemotherapy, GERD, and intermittent asthma. Her medications included crizotinib. She was diagnosed with esophageal stricture and was admitted to the hospital for balloon dilatation. Shortly after the procedure though, she became hypotensive, tachycardic, and tachypneic, and she was admitted to the ICU.

Chest X-ray at this time demonstrated new right-sided infiltrates, thought to be secondary to aspiration pneumonia, and the patient was started on vancomycin and meropenem. There was also a concern for esophageal perforation, so a CT scan was performed, and it revealed a large right posterior thoracic mass with a mediastinal invasion that was obstructing the right pulmonary artery, in addition to an enlarged right heart. An echocardiogram that was also done showed right ventricular dilation, along with severe pulmonary hypertension, with a pulmonary artery systolic pressure of 75-80 mmHg. 

 Despite optimal management, the patient continued to be tachypneic, hypoxemic, and dyspneic. As her respiratory status continued to decline, the patient was intubated, and the family decided to have a ‘do not resuscitate’ order for the patient. During her stay in the ICU, the patient became consistently tachycardic with a heart rate ranging from 100 to 120 beats per minute. Given her respiratory failure, active malignancy, and prolonged immobilization, the patient’s Geneva score was calculated to be 9, and a CT angiography (CTA) of the chest was ordered to rule out pulmonary embolism.

**Figure 1:**
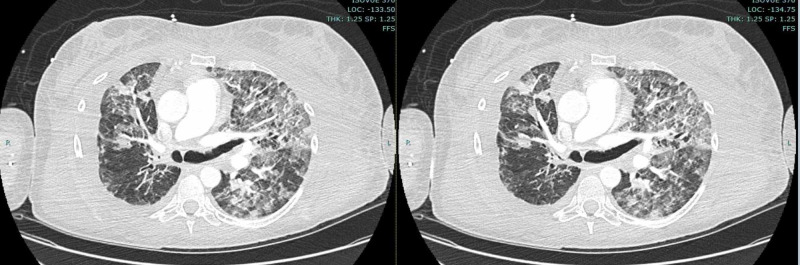
CTA revealed concentric narrowing of the main right pulmonary artery, with pulmonary artery occlusion to the right lower lobe. CTA, CT angiography

**Figure 2:**
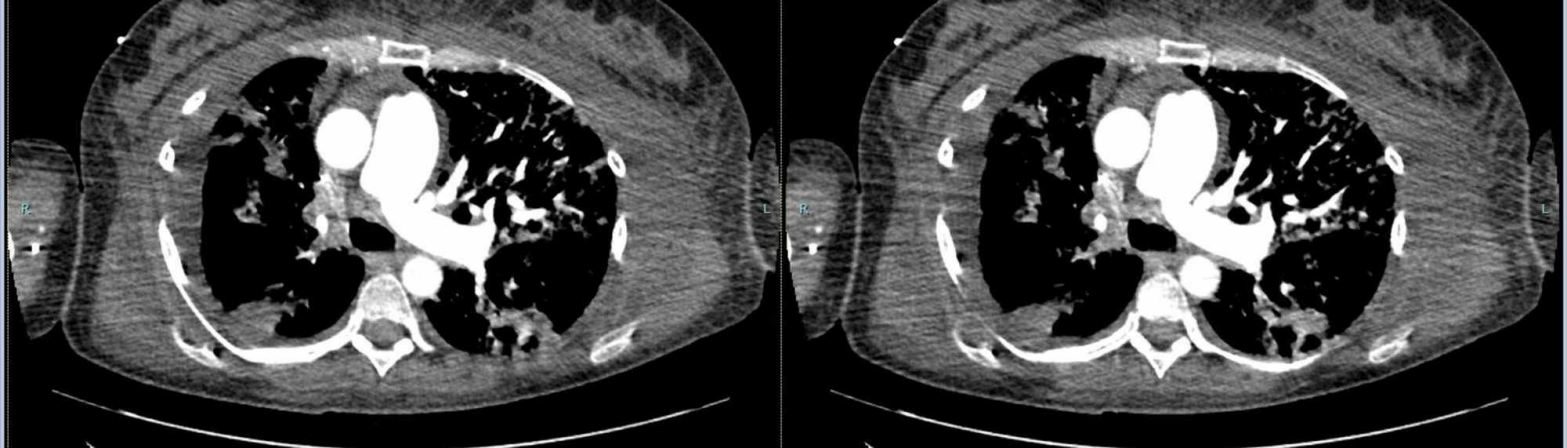
CTA revealed concentric narrowing of the main right pulmonary artery, with pulmonary artery occlusion to the right lower lobe. CTA, CT angiography

**Figure 3:**
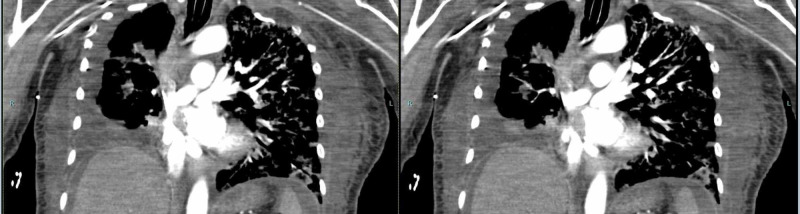
CTA revealed concentric narrowing of the main right pulmonary artery, with pulmonary artery occlusion to the right lower lobe. CTA, CT angiography

**Figure 4:**
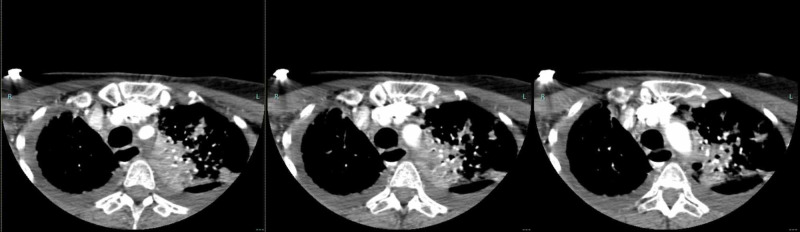
CTA revealed concentric narrowing of the main right pulmonary artery, with pulmonary artery occlusion to the right lower lobe. CTA, CT angiography

CT angiography showed filling defect and concentric narrowing of the main right pulmonary artery, with pulmonary artery occlusion to the right lower lobe, likely due to arterial embolus or mass. The scan also showed invasion of the left atrium, as well as invasion of the left and right pulmonary veins (Figures 1-4). The patient’s clinical status continued to worsen, and she was treated for distributive/cardiogenic shock with maximal blood pressure support, but she, unfortunately, succumbed to death. The time elapsed from admission to time of death was three weeks.

## Discussion

The initial insult in tumoral pulmonary hypertension is typically neoplastic microemboli obstructing the distal pulmonary vasculature, causing pulmonary microvascular disease. However, a rare cause of tumoral pulmonary hypertension is tumor macroembolism that affects the proximal pulmonary arteries. This disease process, known as PTE, is rarely seen in patients with end-stage malignancy. In one report that studied autopsies from 65,181 cancer patients, the incidence of PTE was found to be 0.19% [3].

 There are three different mechanisms thought to be behind the pathogenesis of PTE. The first involves a primary tumor in a distant organ that embolizes to the bloodstream, where it can cause occlusion of the pulmonary arteries without invasion [4-5]. Another mechanism is caused by tumor cells being spread hematogenously, where the cells will lodge into the pulmonary arteries. This is known as pulmonary tumor thrombotic microangiopathy (PTTM), and it results in thrombus formation within the pulmonary arteries, leading to occlusion of the vessels [6]. Finally, in a primary or secondary lung malignancy, such as in this patient, PTE can be caused by local invasion and occlusion of the pulmonary arteries [7]. 

 Diagnosing PTE antemortem is challenging, as patients will often present with nonspecific symptoms such as progressive dyspnea, pleuritic chest pain, and less commonly cough, hemoptysis or fatigue. Physical exam findings include tachypnea, tachycardia, hypoxemia, and low-grade fever. In around 15%-20% of patients diagnosed with PTE, signs of rapidly progressive signs of right heart failure can be seen, such as jugular venous distension, peripheral edema, or ascites [8-9]. Plain film radiographs are not reliable studies for diagnosing PTE as the disease can look similar to pneumonia or interstitial lung disease [10]. CTA is a good imaging modality for visualizing proximal macroembolisms in large arteries, and scans can show pulmonary artery filling defects [11]. However, CTA cannot reliably differentiate between a thromboembolism and a tumor embolism.

## Conclusions

Unfortunately, treatments for PTE have not been studied well. Some cases have reported using endothelin receptor antagonists like bosentan and ambrisentan in order to slow down the pulmonary vasculature remodeling process. It is also possible that chemotherapy may play a role in decreasing tumor burden in PTE. However, the role of these agents is not clear yet, and further study needs to be done. Prognosis after diagnosis of PTE is poor, and patients usually do not survive longer than a few weeks.
